# Attosecond and nano-Coulomb electron bunches via the Zero Vector Potential mechanism

**DOI:** 10.1038/s41598-024-61041-2

**Published:** 2024-05-11

**Authors:** R. J. L. Timmis, R. W. Paddock, I. Ouatu, J. Lee, S. Howard, E. Atonga, R. T. Ruskov, H. Martin, R. H. W. Wang, R. Aboushelbaya, M. W. von der Leyen, E. Gumbrell, P. A. Norreys

**Affiliations:** 1 Department of Physics, University of Oxford, Oxford, OX1 3PU UK; 2 John Adams Institute for Accelerator Science, University of Oxford, Oxford, OX1 3RH UK; 3 Plasma Physics Department, AWE, Aldermaston, RG7 4PR UK

**Keywords:** Laser-produced plasmas, Plasma-based accelerators

## Abstract

The commissioning of multi-petawatt class laser facilities around the world is gathering pace. One of the primary motivations for these investments is the acceleration of high-quality, low-emittance electron bunches. Here we explore the interaction of a high-intensity femtosecond laser pulse with a mass-limited dense target to produce MeV attosecond electron bunches in transmission and confirm with three-dimensional simulation that such bunches have low emittance and nano-Coulomb charge. We then perform a large parameter scan from non-relativistic laser intensities to the laser-QED regime and from the critical plasma density to beyond solid density to demonstrate that the electron bunch energies and the laser pulse energy absorption into the plasma can be quantitatively described via the Zero Vector Potential mechanism. These results have wide-ranging implications for future particle accelerator science and associated technologies.

## Introduction

Attosecond spectroscopy has taken centre stage, as recognised by the recently awarded Nobel Prize in Physics^1^. Chirped Pulse Amplification, pioneered by Donna Strickland and Gerard Mourou^2^, has ushered in a new era of multi-petawatt class laser facilities. Such facilities are in the processes of construction and commissioning worldwide: in the United States^3,4^, United Kingdom^5^, France^6^, Czech Republic^7^, Romania^8^, China^9–11^, South Korea^12^ and Japan^13,14^, among others^15^. These facilities, capable of providing focused intensities up to and beyond $10^{23}$ W cm^-2^, will provide the opportunity to probe the interaction of fully relativistic laser pulses with overdense plasma on attosecond timescales and including the onset of quantum electrodynamic (QED) effects. For sufficiently smooth solid targets at these destructive laser intensities, the first several cycles of an incident laser pulse give rise to coherent electron motion on the front surface of the target, resulting in high harmonic generation (HHG) in reflection and a train of attosecond electron bunches in transmission, providing new opportunities for the production of attosecond light. Unlike laser-gas interactions, such techniques can take full advantage of the high laser intensities now available to create sources of extreme brightness^16^.

Due to the high complexity of the interaction, it is not possible to construct models ab initio. Instead, phenomenological models are constructed via relation to Particle-In-Cell (PIC) simulations to then be tested in experiments. The first successful description of an intense laser-dense plasma interaction was the Oscillating Mirror Model^17^, then modified for the highly relativistic case^18^. For more details on the early developments see^19^. More recently, the Relativistic Electron Spring^20^, the Coherent Synchrotron Emission^21^ and the Zero Vector Potential (ZVP)^22^ models have been established. These models consider the competing forces of radiation pressure and electrostatic charge separation on electron dynamics, as has been applied for ion acceleration in the hole boring^23^ and light sail regimes^24^.

Supplementary Movie S1 shows the simulated laser-plasma interaction of interest. For a linearly polarised incident laser pulse, electron motion is confined to a plane and therefore the interaction is in essence two-dimensional. A qualitative description of the interaction under the ZVP framework^22^ proceeds as follows. At sufficiently high incident laser pulse intensities, electrons at the front surface of the plasma block are accelerated to relativistic speeds in a fraction of a laser cycle and therefore follow similar trajectories. The electrons are displaced into the plasma bulk via the ponderomotive pressure of the laser pulse, forming a high-density, spatially thin and coherent electron bunch. As the plasma ions are approximately immobile on the timescale of a laser pulse cycle, a positive space charge remains in the wake of the electrons. This charge separation generates a longitudinal electric field, the so-called pseudo-capacitor. Propagation of the electron bunch into the plasma bulk is halted when the ponderomotive pressure of the laser pulse is balanced with the pressure exerted by the electrostatic pseudo-capacitor field. Should the electron momentum transverse to the laser pulse go to zero, the $J\times B$ force will no longer act into the plasma and the instantaneous ponderomotive pressure will vanish. By canonical conservation of transverse momentum, this occurs precisely when the zero of the laser pulse vector potential passes through the electron bunch. Equivalently, this occurs at the peak of the laser pulse electrostatic field. In the absence of ponderomotive pressure, electrons freely accelerate across the pseudocapacitor field, discharging it in the process, and thus electron transverse momentum passing through zero is followed by a dramatic increase in linear momentum away from the plasma surface. Macroscopically, one observes rapid acceleration of the surface away from the plasma bulk. In reality, the electron bunch has some thickness. In general zeros of the vector potential cannot enter a plasma, instead the vector potential decays exponentially within the plasma skin depth and thus zeroes cannot interact with the full electron bunch. However, it has been shown that for a plasma moving at speed *u*, zeros are present in the plasma skin layer but are expelled at speed $c^2/u$^22^. For a short time, therefore, the electron bunch co-propagates with the zero of the vector potential. Concurrently, a sharp pulse of electromagnetic radiation is emitted in reflection. This is known as High Harmonic Generation (HHG). HHG was first experimentally identified using Chirped Pulse Amplification by Norreys et al.^25^. Here, HHG occurs via Coherent Synchrotron Emission (CSE), as first observed by Dromey et al.^26^. Indeed, Cousens et al. identified that CSE occurs when the transverse velocity goes to zero^27^, corresponding to the zero of the vector potential. Thus the models of CSE and ZVP are intrinsically linked, however, while CSE theory focuses on laser pulse reflection at the zero of the vector potential, ZVP theory seeks to understand laser pulse energy absorption into the plasma post zero. Half a laser cycle later, the electron bunch encounters the next peak of the laser pulse and is rotated back towards the plasma bulk and the process repeats, until, with the growth of instabilities, de-coherence and plasma destruction occur.

Bunches are produced at a frequency of $2\omega _\mathrm{L}$, where $\omega _\mathrm{L}$ is the angular frequency of the laser pulse, and form a train that propagates through the plasma bulk in the laser transmission direction. These bunches drive two-stream and filamentation instabilities^28^, as well as plasma waves. Eventually, they escape from the back of the plasma, imprinted by the instabilities. This bunch train is shielded from the laser, however, the presence of a return current in the plasma bulk generates an electric field at the back surface, decelerating the electron bunches as they escape the plasma. On such timescales, collisions are negligible. Most interesting are those electrons a distance less than twice the relativistic Larmor radius, $r_\mathrm{L} = \gamma mv/eB$, from the plasma edge. These electrons escape to the sides of the plasma bulk when they are rotated back towards the plasma by the magnetic field of the subsequent peak of the laser pulse. They are accompanied by a burst of radiation in transmission as can be observed in the $E_y$ field of Supplementary Movie S1. This radiation is imprinted by the properties of the electron bunch: large divergence but attosecond duration. Note that such duration is indicative of a CSE-type harmonic spectrum as is anticipated in transmission from such relativistic laser-plasma surface interactions^29^. The side to which the bunches escape switches every half cycle of the laser pulse as the direction of the $J\times B$ force switches. These bunches are also affected by instabilities in the bulk but to a lesser degree. The bunch trains that escape to the sides continue to be ponderomotively accelerated by the laser after expulsion and have a significantly higher energy density and notably shorter duration. To escape the plasma bulk, the target must be transversely mass-limited relative to the focal spot size of the laser, however, note that coherence of electron motion is reduced with decreasing target size.

In recent years, there has been mounting interest in the production of electron bunch trains from dense plasmas, in part due to the higher charge densities obtainable at lower energy than for gas-density plasmas^30^, and many novel setups have been suggested to produce them^16,31–38^ with some experimental evidence of their existence^30,39,40^. Interest in such electron bunches extends beyond their production. A laser pulse cannot propagate through an overdense plasma, therefore if the laser intensity is sufficiently high that the electrons can respond adiabatically to the $J\times B$ force, these electron bunches provide the dominant laser energy absorption route into the plasma. It is also known that the electron motion is coupled to the HHG^27,41,42^, a phenomenon that spatiotemporally compresses the incident laser pulse, providing a realistic route to the Schwinger Limit^43^ and to X-ray sources that could rival the brightness of current X-FEL facilities^42^.

Considering the vastly differing orders of magnitude associated with petawatt laser pulses, solid density plasmas, micrometre wavelengths and attosecond bunches, dimensionless parameters become exceedingly useful tools. Those relevant to this discussion are the normalised vector potential, $a_0 = eE_\mathrm{L}/(m_\mathrm{e} c \omega _\mathrm{L})$, where $E_\mathrm{L}$ is the peak laser electric field strength, $\omega _\mathrm{L}$ is the laser pulse angular frequency, and *e* and $m_\mathrm{e}$ are the charge and mass of an electron, and the plasma density relative to the critical density, $\bar{n}_\mathrm{e} = n_\mathrm{e}/n_\mathrm{c}$, here $n_\mathrm{e}$ is the electron density and $n_\mathrm{c} = \epsilon _0m_\mathrm{e}\omega _\mathrm{L}^2/e^2$ is the critical density at which the plasma becomes opaque to the laser electric field, assuming relativistic effects can be ignored.

Relativistic similarity theory states that the laser-plasma response does not depend on $\bar{n}_\mathrm{e}$ and $a_0$ independently but via the relativistic similarity parameter

1 $$\begin{aligned} S = \frac{\bar{n}_\mathrm{e}}{a_0}, \end{aligned}$$

accounting for relativistic effects in the overdensity of the plasma. Although not confirmed, previous work on the ZVP mechanism has suggested that the ZVP regime is valid for relativistic interactions, $a_0 > 1$, with $S \ge 1$, while for $S<1$, the onset of relativistically self-induced transparency effects^44^ renders the model invalid. Equally, for large $a_0$, the onset of QED effects should cause the breakdown of relativistic similarity.

Here we present PIC code simulation results in two and three dimensions demonstrating the production of electron bunches via the ZVP mechanism from target edges with low emittance, attosecond duration and high charge density, with the total electron bunch charge theoretically scaling linearly with the laser pulse intensity and focal spot size. These simulations demonstrate the production of an electron bunch with a mean energy of $51 \pm 11$ MeV and duration of 35 as that has a transverse geometric emittance of $35 \pm 7$ nm-rad and a corresponding three-dimensional (3D) simulation predicts an electron bunch with a charge of 9.3 nC can be produced with realistic laser parameters. As electron bunches are typically $\sim$ 1 nC in conventional accelerators, whose electron bunch emittance properties are $\sim$ mm rad before injection into a damping ring, and $\sim$ nm rad post-damping ring for forefront colliders^45,46^, the ZVP electron bunches compare favourably with those conditioned in forefront colliders and open new methods for injection and emittance control. They are therefore ideal candidates both for direct production of bright, hard and attosecond duration coherent X-rays and for injection into secondary accelerators such as laser or plasma wakefield accelerators.

We then show quantitatively that electron bunch mean energies and laser energy absorption into the plasma can be determined by the ZVP mechanism for $10< a_0 < 300$ and relativistic similarity parameter, $S > 1$, and that this mechanism is distinct from the pondermotive acceleration present for circularly polarised laser pulses. Indeed, for $a_0 > 10$, we enter a post-ponderomotive regime of energy absorption, where the plasma density can no longer be neglected in calculations. This has direct implications for HHG and its associated applications: it is well understood that increased $a_0$ most effectively improves the quality of HHG pulses, i.e. increased intensity, reduced duration and increased X-ray content of the produced pulses^20,42,47,48^.

## Results and discussion

**Figure 1:**
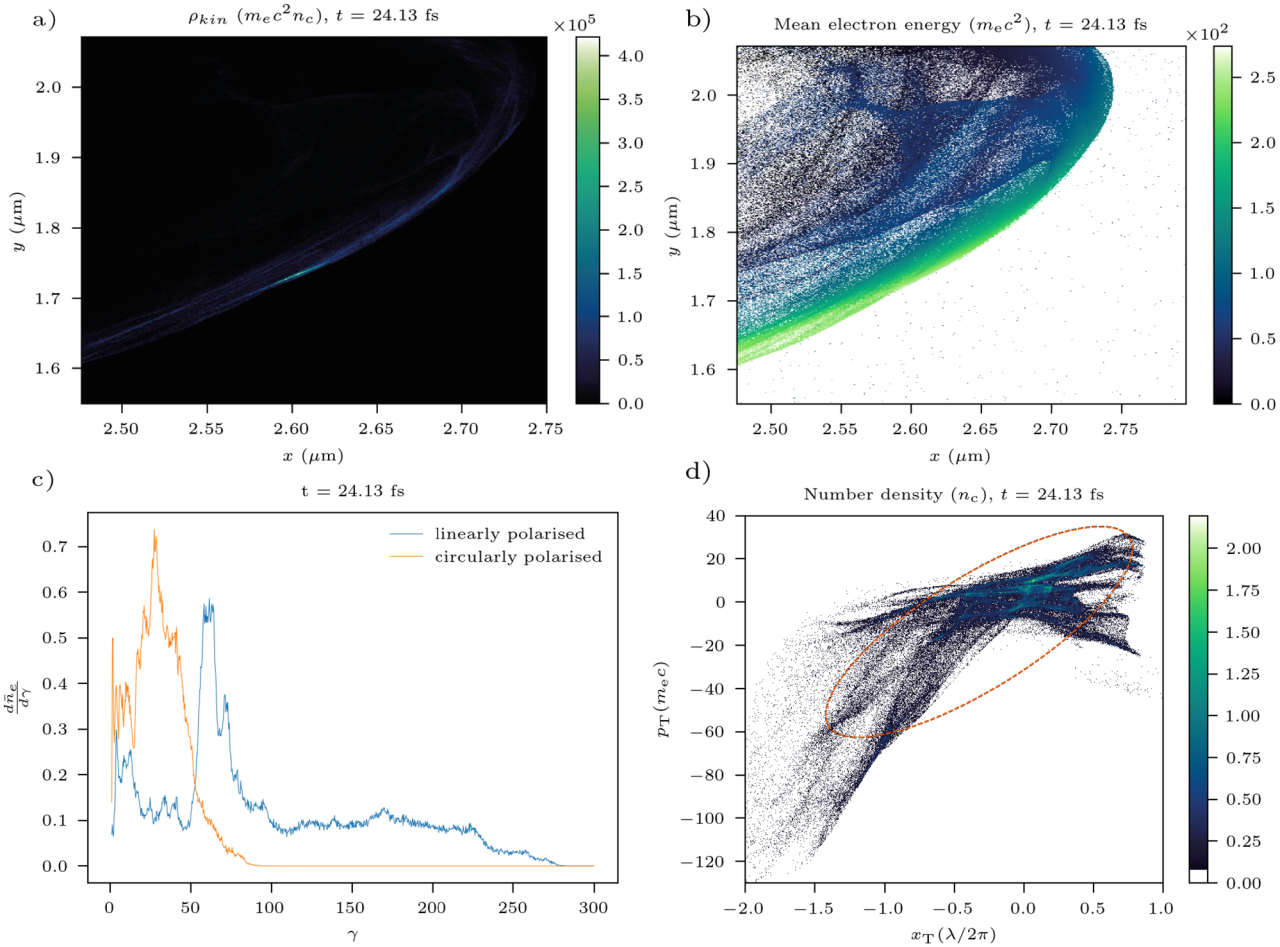
A typical bunch after its expulsion from the plasma edge for the case of ${a_0 = 100}$, ${\bar{n}_\mathrm{e} = 100}$. (**a**) The kinetic energy density. (**b**) The mean electron energy. Points with no data are white. The smooth variation in energy is a product of the quasi-monochromatic nature of the electron bunch as discussed in Ref.^22^. (**c**) The gamma spectrum of the electron bunch compared to that formed by a circularly polarised laser pulse. (**d**) The phase space transverse to bunch propagation in the *x*–*y* plane, namely, ($x_\mathrm{T}$, $p_\mathrm{T}$) as defined in the “Methods” section. Again the dashed line marks the area defining the transverse normalised emittance. The skew of the ellipse is due to a low-density tail on the phase space beyond the bottom left corner of the plot.

To characterise the interaction, the following simulations were performed in 2D. Properties of a typical bunch are presented in Fig. 1. The electron bunch is ultra-relativistic with a mean energy of $51 \pm 11$ MeV and a duration of 35 as. The bunch propagates at an angle of − 0.393 rad to the *x*-axis. The transverse geometric emittance in the *x*–*y* plane is $35 \pm 7$ nm-rad. The transverse emittance is therefore comparable to pre-injectors for state-of-the-art nano-Coulomb electron bunch accelerators^46,49^ but at a significantly higher instantaneous peak current. Intense attosecond X-ray pulses can be produced from such electron bunches via bremsstrahlung in a solid target^50^ or through the interaction with a counter-propagating laser pulse^51^. The shortest XFEL X-ray pulse so far obtained is 280 as^52^. The shorter duration of attosecond electron bunches produced in this mechanism would allow the study of ultra-fast electronic phenomena in matter, applying to the study of a broad range of chemical, physical and biological systems^53^.

**Figure 2:**
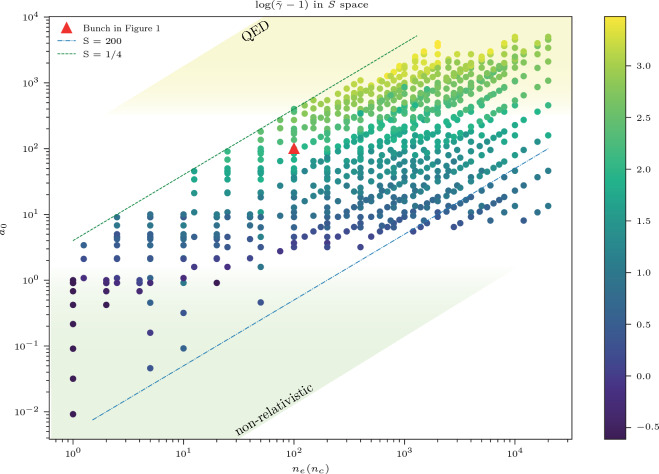
The mean electron bunch energies parameter scan. The energies are extracted from 2D3V Particle-In-Cell (PIC) simulations. On average 8 electron bunches are formed in each simulation, each with a unique $a_0$ due to the Gaussian temporal envelop of the laser pulse. The bunch described in more detail in Fig. 1 is highlighted. The majority of the data points are in the range $0.25< S < 200$ and the transitions in $a_0$ from non-relativistic through to the QED regime are captured.

**Figure 3:**
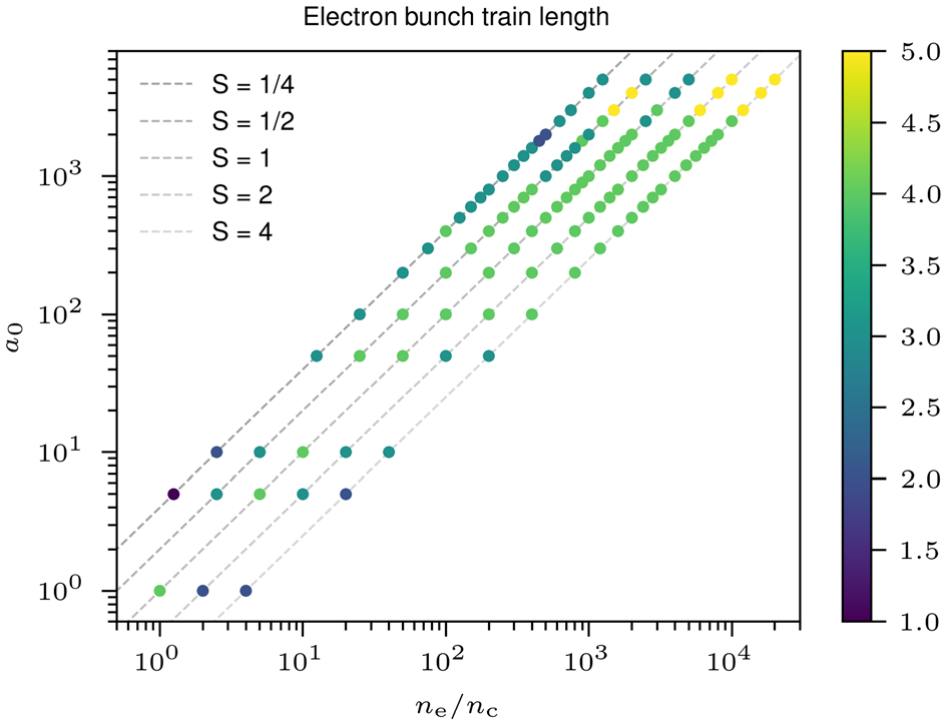
Attosecond electron bunch train length as a function of peak laser pulse normalised vector potential and normalised plasma bulk electron density. In simulations with a peak $S < 1$, the plasma bulk will experience a transition to relativistic self-induced transparency in the rising edge of the laser pulse, thus leading to a breakdown in the ZVP mechanism and an early cut off to the attosecond electron bunch train.

To demonstrate the link between the electron bunches and the ZVP mechanism, Fig. 1c shows the distinctly different energy spectrum of the electron bunch compared to that created with a circularly polarised laser pulse but in all other regards equivalent simulation. The mean energy is over three times lower and there is no quasi-monochromatic nature to the spectrum^22^. For a circularly polarised laser pulse, electrons are continuously ejected from the plasma forming a corkscrew-type bunching structure. At no point do zeros of the vector potential pass through the electron bunches, and therefore there can be no ZVP acceleration phase. To test the ZVP model and to determine for what parameter space it is valid, 120 2D simulations were performed. Mean energies of electron bunches escaping from the top and bottom of the plasma were recorded at two windows, one centred at (${2.53}\;{\upmu {\hbox {m}}}$, ${1.81}\;{\upmu {\hbox {m}}}$) and one at (${2.53}\;{\upmu {\hbox {m}}}$, ${8.72}\;{\upmu {\hbox {m}}}$), each of size (${0.10}\;{\upmu {\hbox {m}}}$ $\times$ ${0.084}\;{\upmu {\hbox {m}}}$). These window positions are asymmetrical relative to the target edges from which the electron bunches are expelled, at (${2.12}\;{\upmu {\hbox {m}}}$, ${2.12}\;{\upmu {\hbox {m}}}$) and (${2.12}\;{\upmu {\hbox {m}}}$, ${8.48}\;{\upmu {\hbox {m}}}$), respectively. Electrons in both these windows should experience the same ZVP acceleration and corresponding gain in energy. However, there will be a small difference in energy from a secondary acceleration phase after expulsion from the target, discussed in more detail in the following section. This discrepancy is small relative to the parameter space explored and therefore mean electron bunch gamma factors from both diagnostic windows are plotted in Fig. 2. In each simulation, around 8 bunches are produced before the breakdown of the plasma, four to each side, for a total of 856 data points. The bunch train length as a function of the laser peak intensity and plasma density for each simulation is plotted in Fig. 3. The bunch number can be reduced by decreasing the peak *S* for the material suggesting a route to isolated attosecond electron bunches. This can be understood from the transition in laser intensity on the rising edge of the laser pulse to relativistic transparency, placing an early cutoff to the bunch production mechanism. Given the intrinsic link between CSE and ZVP electron bunch generation, it is likely that many techniques for isolated attosecond radiation generation can also generate isolated attosecond electron bunches. Possible techniques include few-cycle laser pulses^54^, the attosecond lighthouse technique^55^ using laser pulse wavefront rotation, non-collinear laser pulse gating^56^ or circular polarisation gating^29^.

### The ZVP model

Following the theory presented in Ref.^22^ and updated for 2D by Ref.^41^, relations for the electron bunch mean and total energy can be derived in terms of the laser intensity and plasma density as follows. Note that throughout the electron bunch is treated as infinitesimally thin. This has proven to be a reasonable assumption in previous work^22,41^. Sub-bunch dynamics are explored in detail by Gonoskov et al.^57^.

Consider a plasma block of density $n_\mathrm{e}$ irradiated by a laser pulse with wavelength $\lambda$ and peak electric field $E_\mathrm{L}$. If the electron fluid is displaced a small distance $\Delta \textbf{x}$ by a laser pulse, then the total charge displaced is $Q = -en_\mathrm{e}\sigma |\Delta \textbf{x}|$, where $\sigma$ is the surface area of the interaction. From Gauss’ Law, the field of an appropriately aligned capacitor with charge $\pm Q$ on the plates is $\mathbf {E_\mathrm{C}} = (-Q/\epsilon _0\sigma ){\hat{\mathbf{x}}}$, where $\hat{\mathbf {x}}$ is the unit vector in the positive *x* direction. The pressure exerted by this field on the electron bunch is $\mathbf {P_\mathrm{C}} =Q\mathbf {E_\mathrm{C}}/\sigma$. At peak displacement this is equal and opposite to the peak instantaneous pondermotive pressure, $\mathbf {P_\mathrm{L}} = \epsilon _0 E_\mathrm{L}^2{\hat{\mathbf{x}}}= \epsilon _0(a_0\omega _0m_\mathrm{e}c/e)^2{\hat{\mathbf{x}}}$. Equating the magnitudes of $\mathbf {P_\mathrm{L}}$ and $\mathbf {P_\mathrm{C}}$, the maximum displacement of the electrons is then

2 $$\begin{aligned} \Delta \textbf{x} = \frac{\lambda }{2\pi } \hspace{0.4mm} \frac{a_0}{\bar{n}_\mathrm{e}}{\hat{\mathbf{x}}}= \frac{1}{kS} {\hat{\mathbf{x}}}, \end{aligned}$$

where *k* is the wave vector of the laser pulse. Hence, the peak pseudo-capacitor field is

3 $$\begin{aligned} \mathbf {E_\mathrm{C}} =\frac{en_\mathrm{e}}{\epsilon _0}\Delta \textbf{x} = \frac{\omega _0 cm_\mathrm{e}a_0}{e}{\hat{\mathbf{x}}}= E_\mathrm{L} {\hat{\mathbf{x}}}. \end{aligned}$$

Using the results of Eqs. (2) and (3), the energy, *T*, gained by a single electron launched from the plasma surface is

4 $$\begin{aligned} T = e\mathbf {E_\mathrm{C}}\cdot \Delta \textbf{x}= m_\mathrm{e} c^2 \hspace{0.4mm} \frac{a_0^2}{\bar{n}_\mathrm{e}}, \end{aligned}$$

which corresponds to an electron gamma factor, $\gamma = 1/\sqrt{1 - \beta ^2} = 1 + a_0^2/\bar{n}_\mathrm{e}$. The total energy of the bunch, *U*, is

5 $$\begin{aligned} U = n_\mathrm{e}\sigma |\Delta \textbf{x}|T = \frac{\sigma n_\mathrm{c}}{k} \times m_\mathrm{e} c^2 \hspace{0.4mm} \frac{a_0^3}{\bar{n}_\mathrm{e}}. \end{aligned}$$

The total number of electrons in the bunch, $N_\mathrm{b}$ that escape to the plasma sides scales as

6 $$\begin{aligned} N_\mathrm{b} = n_\mathrm{e}r_\mathrm{L}|\Delta \textbf{x}|L_z \sim (a_0^2/n_\mathrm{e})L_z, \end{aligned}$$

where $L_z$ is the beam width in the *z*-direction and $r_\mathrm{L}$ is the relativistic Larmor radius, as defined in the introduction.

After expulsion from the plasma edge at the peak of the subsequent laser pulse cycle, electrons experience direct ponderomotive acceleration in the vacuum. Electrons are injected into the field with velocities close to the speed of light and transverse velocities aligned to the electric field in which they accelerate. Those injected close to the axis of propagation can therefore exit the field without undergoing multiple oscillations. This is Vacuum Laser Acceleration^39^, a process which has gained significant attention due to its ability to provide accelerating fields of the order of ${{\hbox {TV}}\,{\hbox {m}}^{-1}}$ across the Rayleigh length of a laser pulse.

While Vacuum Laser Acceleration is described in great detail elsewhere and typically cannot be calculated analytically^39^, in the interest of extending the simple model presented here for comparison to the energies extracted from the simulations, consider the following. The electron bunch travels with the subsequent laser pulse peak. Electrons diverge outwards from the ejection point at the plasma surface. They are initially ejected in phase with the electric field peak amplitude of the subsequent laser pulse cycle, hence near to focus an electron experiences an electric field,

7 $$\begin{aligned} E_y(x,y,t) = E_0 e^{-(y-f_y)^2/L_0^2}\cos (k_\mathrm{L}(x-f_x) - \omega _\mathrm{L}t)= E_0 g(x,y,t). \end{aligned}$$

Since the electric field and transverse velocity are always aligned at ejection, electron energy increases and the work done by the field on the electron is

8 $$\begin{aligned} \Delta T = \int \textbf{F} \cdot \textrm{d}\textbf{x} = e\int E_y(x,y,t) dy, \end{aligned}$$

Electron trajectories are approximately linear from ejection point ($y_\mathrm{e},x_\mathrm{e}$) to observation point ($y',x'$),

9 $$\begin{aligned} x = y\frac{(x'-x_\mathrm{e})}{(y'-y_\mathrm{e})}, \ t = \sqrt{x^2 + y^2}/c. \end{aligned}$$

Now Eq. (8) can be integrated and the increase in the mean gamma factor of the electron bunch is therefore

10 $$\begin{aligned} \Delta \gamma = \int \frac{eE_y(y)\textrm{d}y}{m_\mathrm{e}c^2} = a_0'(y)G, \end{aligned}$$

where $a_0'$ is the peak vector potential of the subsequent laser pulse cycle and

11 $$\begin{aligned} G = \frac{2\pi }{\lambda }\int g(y)\textrm{d}y \end{aligned}$$

with *g*(*y*) as defined in Eq. (7). The ZVP energy gain is fixed by the laser pulse electric field peak at the plasma corner,

12 $$\begin{aligned} \Delta \gamma _\mathrm{ZVP} = \frac{(a_0 e^{-(2\lambda -y_\mathrm{f})^2/L_0^2})^2}{\bar{n}_\mathrm{e}} = 0.61 \frac{a_0^2}{\bar{n}_\mathrm{e}}. \end{aligned}$$

At measurement,

13 $$\begin{aligned} \gamma = 1 + (0.61)\times \frac{a_0^2}{\bar{n}_\mathrm{e}} + G\times a_0'. \end{aligned}$$

The final term of Eq. (13) could be reduced or neglected by the use of a suitable super-Gaussian spatial laser profile or by the use of a plasma separator^58^, a secondary plasma to screen the electromagnetic fields as applied in Ref.^59^.

Using the Ordinary Least Squares regression model provided by the statsmodels Python module^60^, Eq. (13) was fit to the two data sets, allowing the pre-factors to vary freely. The linear model can be applied to the non-linear relationship by constructing the necessary composite parameter for the ZVP energy. The fits have $r^2$-values of 0.81 and 0.84 respectively. Reassuringly, the two fits find the same ZVP acceleration pre-factor, $G_\mathrm{ZVP} = 0.47 \pm 0.02$, slightly lower than the ideal energy calculated from the model, a likely consequence of the finite width of the pseudocapacitor.

The first data set was extracted a distance (${0.41}\;{\upmu {\hbox {m}}}$, ${0.25}\;{\upmu {\hbox {m}}}$) from the target edge, corresponding to $G = 0.31$ compared to $0.22 \pm 0.02$ predicted by the fit. The second, extracted a distance (${0.41}\;{\upmu {\hbox {m}}}$, ${0.31}\;{\upmu {\hbox {m}}}$) from the target edge, corresponds to $G = 0.39$ compared to $0.34 \pm 0.02$ predicted by the fit.

**Figure 4:**
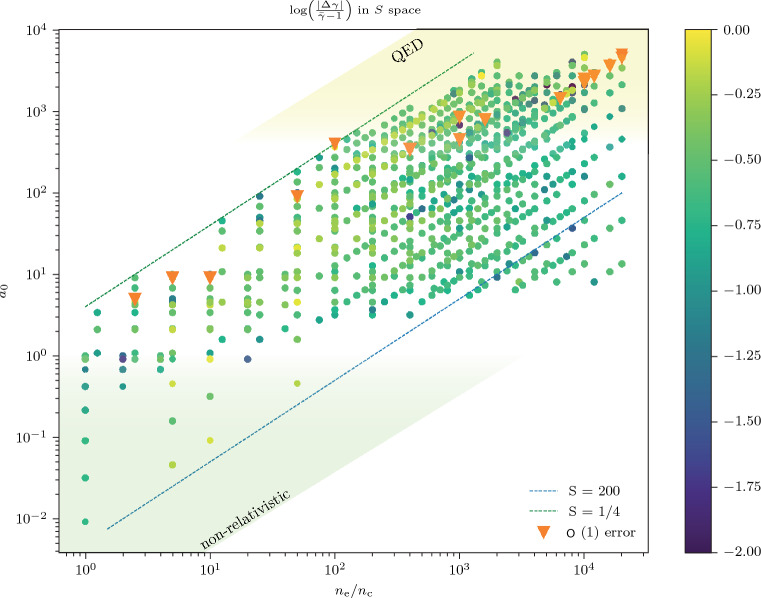
A log plot of the errors associated with the data of Fig. 2, when compared to the model. The orange triangles represent a failure of the model to predict the electron bunch mean energy. The best fit to the model is for the range $10 \le a_0 \le 300$ and $S \ge 1$.

Figure 4 compares the relative errors on all data points. Errors greater than an order of magnitude are marked with orange triangles. Reassuringly these anomalous points appear solely in the QED regime and for $S<1$, where relativistic effects must be accounted for. The model’s success is inconsistent in the non-relativistic regime as can be expected from the model assumptions and there is no indication that large *S* leads to a breakdown of the model.

In the original ZVP theory paper, Baeva et al.^22^ state $a_0 \gg 1$ and $S > 1$ and present simulation results exploring the range $S \le 10$ and $a_0 \le 40$. Savin et al.^41^ then explored the range $S \le 10$ and $a_0 \le 100$ in simulations. In the 2019 paper from Savin et al.^61^, the relativistically underdense transition region is probed in simulation, i.e., $S \le 1$, although it was noted in this work that radiation reaction can suppress relativistic transparency. The simulations presented here explore a significantly larger parameter space. This is in part now a practical endeavour due to the new strategy of the extraction of multiple electron bunches from each simulation enabling access to high *S* values from early laser pulse cycles. Thus, it would appear that the region of validity of the model extends further than was previously considered, opening up this field to a wider range of scenarios, such as the case of shock-compressed plasmas.

### Energy absorption in the ZVP regime

**Figure 5:**
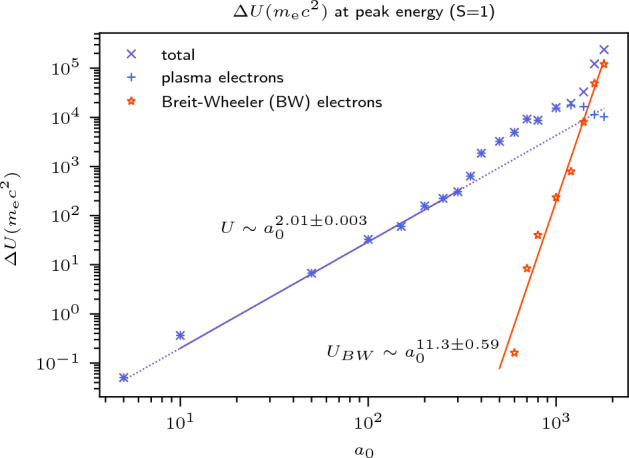
The peak instantaneous total energy of electron bunches escaping to the rear of the plasma bulk, with constant *S*. The ZVP mechanism predicts that the electron bunch total energies and therefore laser energy absorption should scale as $U \sim a_0^2$ for constant *S*. Those electrons formed via the Breit–Wheeler pair production process begin to dominate for $a_0 > 1000$.

As the ZVP mechanism is the dominant mode for energy absorption by the plasma, Eq. (5) describes the scaling of energy absorption in the bulk. Since electron bunches are generated twice per laser period, the rate of energy absorption, $R = 2\times U(\omega _\mathrm{L}/2\pi ) = U\omega _\mathrm{L}/\pi$. Peak instantaneous bunch energies escaping from the back side of the plasma are plotted in Fig. 5.

As *S* was kept constant for these simulations,

14 $$\begin{aligned} U \sim \frac{a_0^2}{S} \sim a_0^2. \end{aligned}$$

A fit to the data within the established range of ZVP validity finds $U \sim a_0^{2.01\pm 0.003}$. In this first treatment of the total energy of the escaping bunches, only the scaling has been considered. Energy transfer from the bunches into the bulk must be taken into consideration such as to the instabilities and to the electric field that forms at the rear of the plasma due to the return current of electrons. Figure 5 also shows the energy of the Breit–Wheeler pair produced electrons, which begin to dominate the total bunch energies at $a_0 \approx 1000$. The sharp increase in the total energy absorption at this point and deviation from Eq. (14) could be related to the sudden deviation of mean hot electron temperature above the scaling of Eq. (4) at $a_0 \approx 350$ after the onset of Breit–Wheeler pair production as identified in simulations by Savin et al.^61^. Relativistic ZVP electrons accelerating across the pseudocapacitor field and towards the incident laser pulse can experience significantly boosted electromagnetic fields in their rest frame, enabling the onset of radiation reaction produced photons for fields below the Schwinger Field Limit, $E_\mathrm{S} = {1.32\times 10^{18}}\; {{\hbox {V}} \; {\hbox {m}}^{-1}}$ in the laboratory frame. These high-energy photons, typically with momenta and energies on the order of the electron that made them, can then interact with the laser pulse field via multi-photon Breit–Wheeler pair production. The rate of pair production depends on the photon quantum parameter^62^

15 $$\begin{aligned} \upchi _\gamma = \frac{\gamma _\gamma }{E_\mathrm{S}}\sqrt{(\mathbf {E_\perp } + \textbf{c}\times \textbf{B})^2 - (\textbf{c}\cdot \textbf{E})^2/c^2}, \end{aligned}$$

where $\gamma _\gamma = \hbar \omega _\gamma /m_\mathrm{e}c^2$ is the normalised photon energy, *c* is the photon velocity and $E_\perp$ the electric field perpendicular to the photon. Assuming the electron undergoing radiation reaction radiates approximately all of its ZVP produced energy then $\gamma _\gamma \approx a_0^2/\bar{n}_\mathrm{e}$. At emission, $\sqrt{(\mathbf {E_\perp } + \textbf{c}\times \textbf{B})^2 - (\textbf{c}\cdot \textbf{E})^2/c^2} \sim \sqrt{2}a_0$. Pair production begins to rise rapidly around $\upchi _\gamma = 1$, thus Breit–Wheeler pair production becomes significant when the laser pulse normalised vector potential rises to

16 $$\begin{aligned} a_0 \approx \left( \frac{a_\mathrm{S}\bar{n}_\mathrm{e}}{\sqrt{2}}\right) ^{\frac{1}{3}}, \end{aligned}$$

where $a_\mathrm{S} = {7.73 \times 10^{5}}$ is the normalised vector potential of the laser pulse associated Schwinger Field. Recalculating as a function of *S*, $\gamma _\gamma \approx a_0/S$ and thus

17 $$\begin{aligned} a_0 \approx \left( \frac{a_\mathrm{S}S}{\sqrt{2}}\right) ^{\frac{1}{2}}. \end{aligned}$$

In the work of Savin et al. where $\bar{n}_\mathrm{e} = 50$, this corresponds to $a_0 \approx 301$, whereas here, where $S = 1$, we anticipate the onset of pair production at $a_0 \approx 739$. This is consistent with Fig. 5 and justifies the observed higher intensity of the transition to greater energy absorption compared to the work of Savin et al. Note that the relativistic $\textbf{J}\times \textbf{B}$ scaling for hot electrons, $\gamma \sim \sqrt{1 + a_0^2}$, derived by Wilks et al.^63^ could not predict the lower transition observed by Savin et al.

### The ZVP interaction in 3D PIC simulation

**Figure 6:**
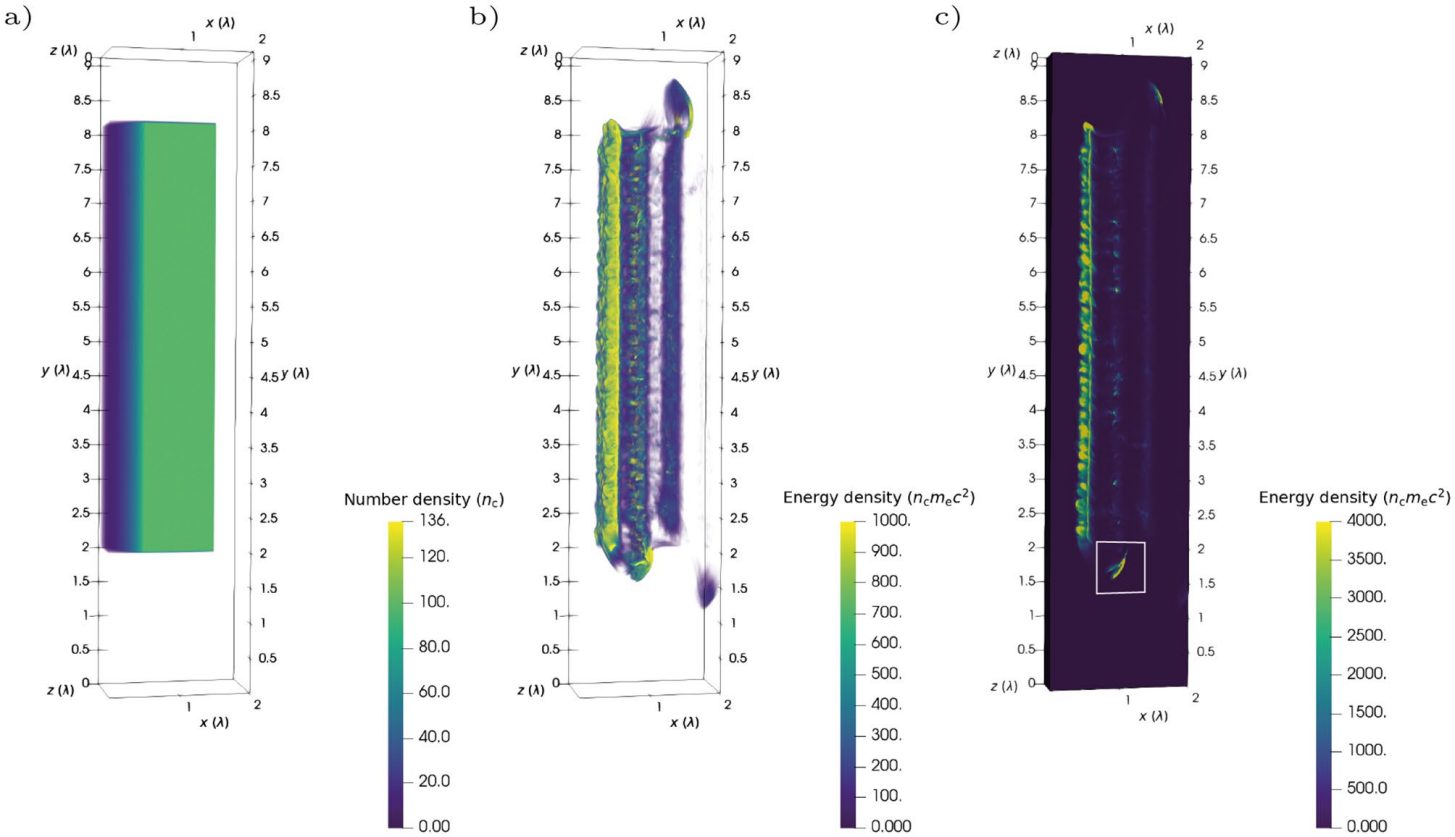
The Zero Vector Potential (ZVP) mechanism in 3D Particle-In-Cell (PIC) simulation with ${a_0 = 100}$, ${\bar{n}_\mathrm{e}}$ = 100. (**a**) Initial electron density, the laser propagates in the $\hat{\mathbf {x}}$-direction. (**b**) Electron kinetic energy density after a few laser cycles. Three electron bunches are visible propagating in the *x*-direction through and around the plasma bulk. The repeating structure on the electron bunches propagating through the plasma bulk is a result of the two-stream instabilities. (**c**) A cross-section through the centre of (**b**) along $z=\lambda /2$, for clearer examination of the internal structure of the plasma block. The bunch referred to in the text is indicated by a white box.

The 3D PIC simulation of a linearly polarised relativistic laser pulse incident on an overdense plasma is presented in Fig. 6. The characteristic formation of both pseudo-capacitor field and energetic high-charge attosecond electron bunches was observed. An equivalent two-dimensional (2D) simulation obtained similar results. The highlighted bunch has a total charge of 0.35 nC, but note the thin width of the plasma in the *z*-direction. For a laser pulse linearly polarised along *y* and propagating along *x*, the forces on the plasma electrons confine electron dynamics to the *x*–*y* plane. There is therefore flexibility in the choice of plasma block thickness in the *z*-direction and thus one can extrapolate: for a realistic laser pulse with beam width 10 $\lambda _\mathrm{L}$ incident on a larger plasma block, a bunch of charge $2\times 10/0.75 \times 0.35 = 9.3$ nC is obtained.

**Figure 7:**
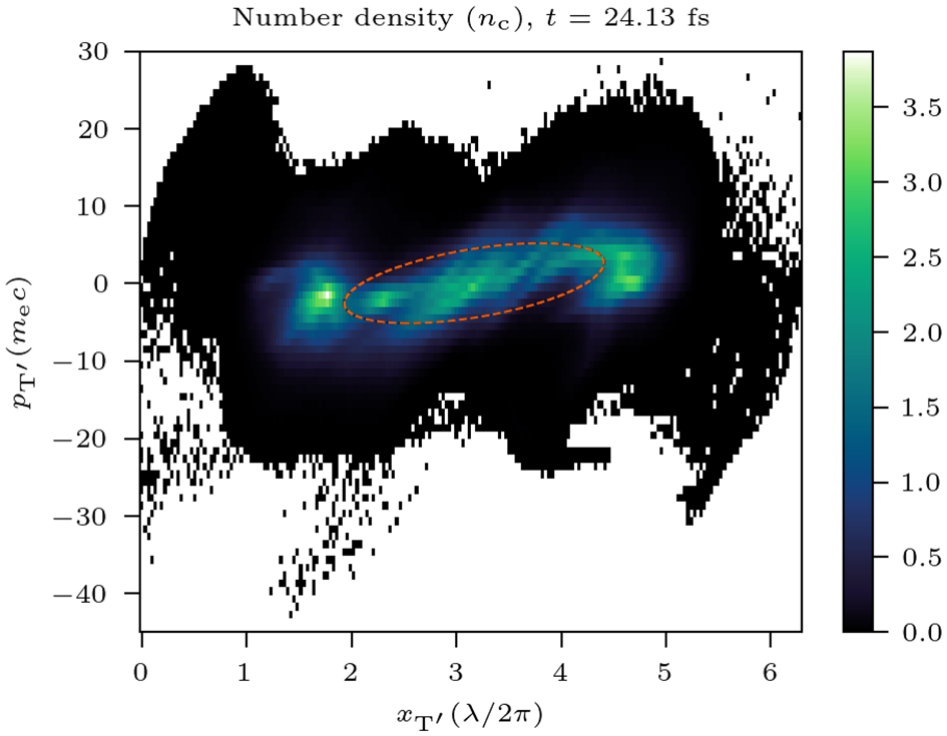
The *z*-${p_z}$ phase space for the 3D simulation, namely, (${x_\mathrm {T'}}$, ${p_\mathrm {T'}}$) as defined in the “Methods” section. The ellipse marked with a dashed line is defined by the relevant Courant–Snyder parameters for the distribution and its area is proportional to the transverse normalised emittance of the electron bunch.

Provided the laser intensity remains relativistic, there is no limit to the size of the plasma bulk and laser spot size in this dimension and therefore on the size of the electron bunch. And so, by doubling the laser pulse energy, one can double the electron bunch maximum obtainable charge. The *z*–$p_z$ phase space of the bunch is presented in Fig. 7. The *z*–$p_z$ transverse geometric emittance of this bunch is $7.4 \pm 1$ nm-rad.

**Figure 8:**
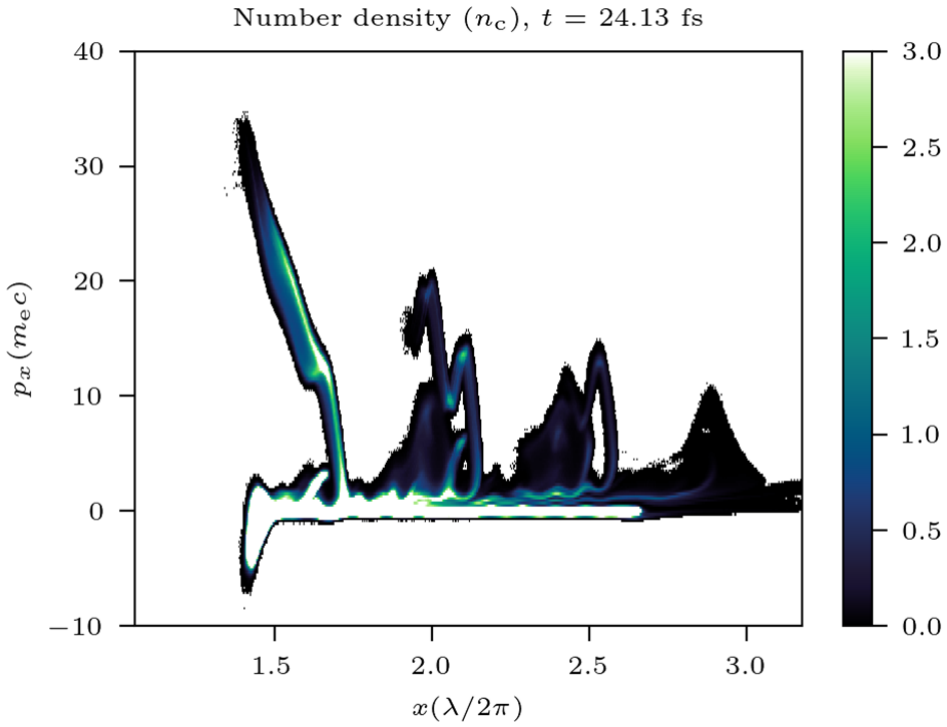
The *x*-${p_x}$ phase space of bunches propagating through the plasma bulk in 3D simulation. Thus, attosecond bunch duration in the spectro-temporal domain is highlighted. The variation in $p_x$ for each bunch is reflective of the Gaussian temporal envelope of the incident laser pulse.

Electron bunches escaping to the sides are of much shorter duration and higher density compared to those bulk propagating electron bunches. The ZVP electron bunch consists of a spectrum of energies. When encountering the subsequent peak of the laser pulse, lower energy electrons are turned back towards the bulk before higher energy electrons. As all electrons travel at approximately *c*, higher energy electrons do not overtake lower energy ones. This led Baeva et al. to describe the quasimonoenergeticity of the electron bunch: the electron bunch is now of attosecond duration in the spectro-temporal domain as demonstrated by the longitudinal phase space plot of the plasma bulk in Fig. 8, extracted from the 3D PIC simulation. At the target edges electron bunches retain their attosecond duration with the trade-off of increased divergence.

**Figure 9:**
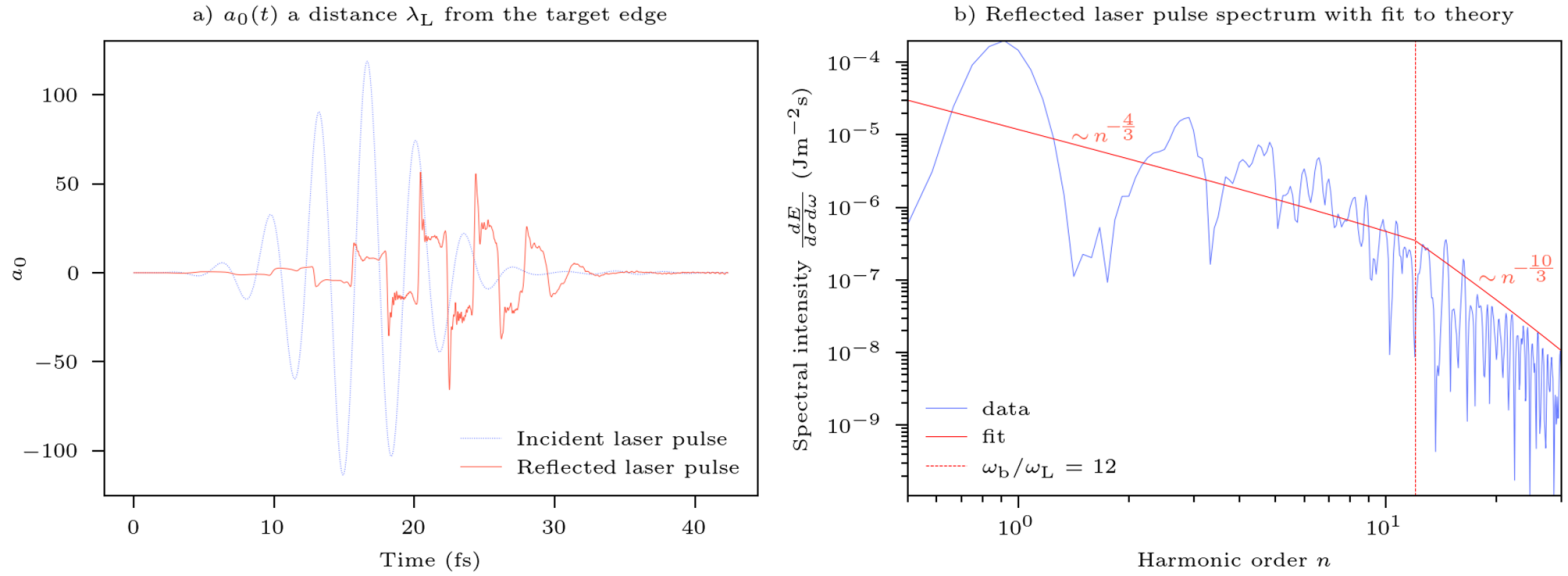
Electric field temporal structure in 3D Particle-In-Cell (PIC) simulation with ${a_0 = 100}$, ${\bar{n}_\mathrm{e}}$ = 100. (**a**) Temporal variation of the normalised vector potential of the incident and reflected laser pulses along the polarisation axis of the incident laser pulse. The reflected pulse demonstrates attosecond radiation spikes without the need for spectral filtering. (**b**) The spectral intensity of the reflected radiation obtained via a Fourier transform of the pulse in (**a**). The fit is calculated following the methodology of Edwards and Mikhailova^42^: $\omega _\mathrm{b}/\omega _\mathrm{L}$ defines the cut-off above which an ordinary least squares fit to $\sim n^{-p}$ yields an exponent, $p > 4/3$. Beyond the cut-off, the spectrum is predicted to scale as $\sim n^{-10/3}$. The fit is a simple weighted polynomial fit to the logarithm of the data using the NumPy polyfit module.

Figure 9a compares the incident laser pulse to the strongly modulated reflected pulse in the 3D PIC simulation. The Fourier transform of the reflected pulse is presented in Fig. 9b. Due to the high laser pulse intensities in these simulations, the spectrum is of the modified CSE type detailed by Edwards and Mikhailova^42^: initially the spectral intensity scales as $\sim n^{-4/3}$ up to a cut-off determined by the advance time bunch width of radiating electrons after which it scales as $\sim n^{-10/3}$. Edwards and Mikhailova demonstrated that this cut-off, extracted from the internal dynamics of the system can be well approximated by the point where the fit to the spectrum drops below the $\sim n^{-4/3}$ scaling, at harmonic number, $n = 12$ in this simulation. This 3D simulation result is consistent with the $n = 11.3$ determined by Edwards and Mikhailova in their most similar 1D simulation at $a_0 =100$, $\bar{n}_\mathrm{e} = 90$, $\theta =$ ${45}^\circ$. It is also interesting that since their definition of the bunch width corresponds to the temporal width of the radiation spike at observation, taking the full-width-half-maximum of the CSE type spikes (between $t = 15$ and 26 fs) in Fig. 9a as the cut off harmonic for each spike gives a mean harmonic cut off of $n = 11.4$ consistent with the spectrum fit and corresponding to an average pulse duration of 292 as. Hence, the cut-off can infer the attosecond pulse duration from a simple UV spectrometer measurement without the need for complex attosecond resolution diagnostics. A second cut-off, dependent on the peak gamma factor of radiating electrons and beyond which the spectrum decays exponentially, is not captured at this simulation resolution. The deviation of the spectrum from regularly spaced harmonics is a natural consequence of the high laser pulse intensity: the non-negligible hole boring velocity (scaling linearly with the electric field strength of the laser pulse^64^) significantly lengthens the path of the reflected pulse, Doppler shifting harmonics between successive pulse cycles.

### Experimental considerations

**Figure 10:**
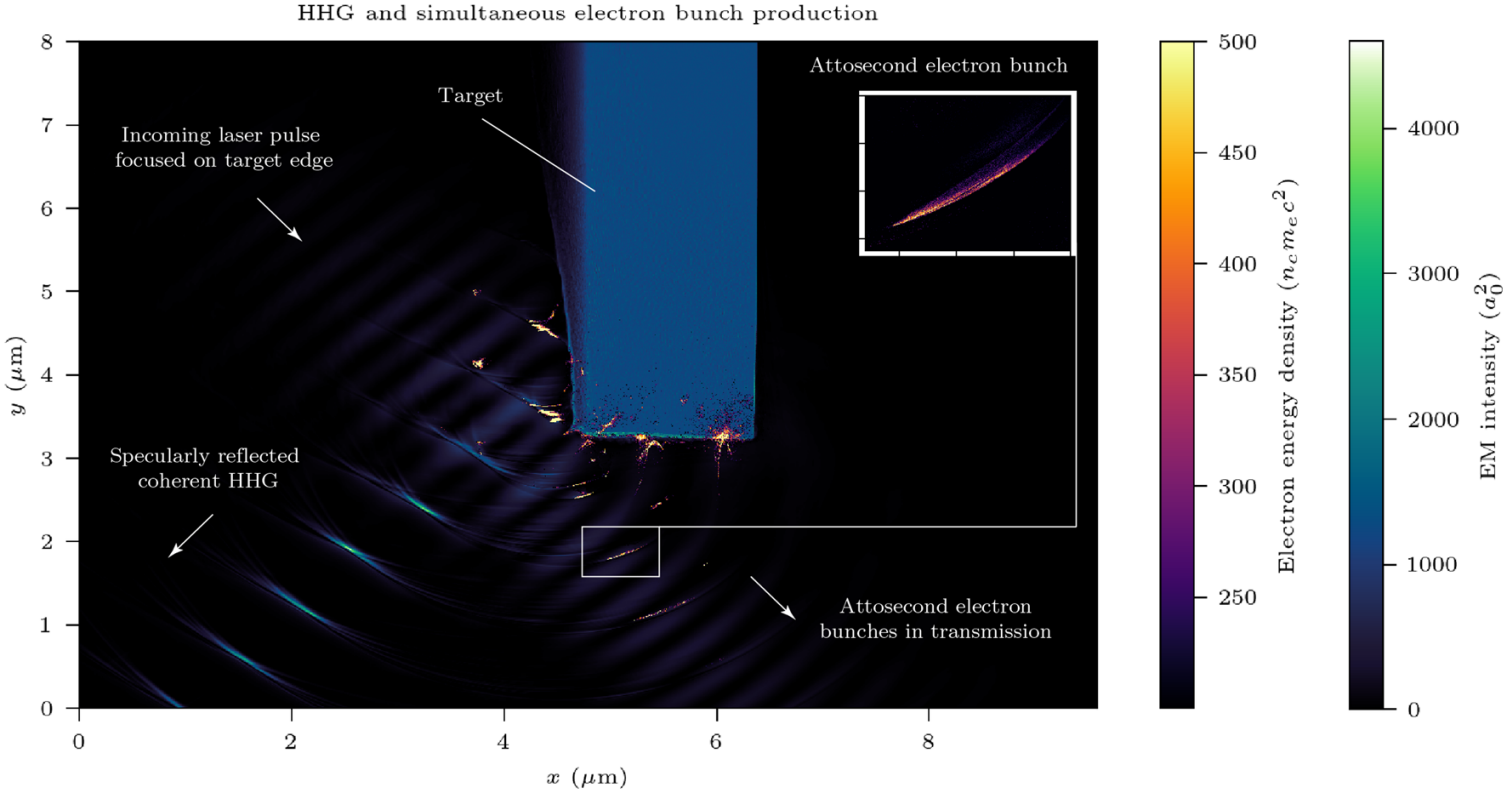
One possible experimental setup. A relativistically intense, $a_0 = 20$, 30 fs laser pulse focused at 45 degrees onto one edge of a low-density polyethylene target with an exponential preplasma of scale length $0.2\lambda _\mathrm{L}$. The inset shows an electron bunch at greater magnification. Attosecond HHG and electron bunches are produced at a rate of $\omega _\mathrm{L}$ due to the oblique incidence angle. The thickness of the target in the $\textbf{x}$-direction is chosen for computational efficiency and does not impact the mechanism.

We now discuss the feasibility of observing the attosecond ZVP electron bunches. The requirements are in reality relatively straightforward: a relativistically intense ($a_0 > 10$) short pulse (tens of femtoseconds) laser, a low-density solid target edge, for example, plastic, and a sharp vacuum-plasma boundary. Prepulse control using plasma mirrors is essential to tailor the preplasma expansion from the intrinsic laser prepulse, as was performed in the experiment of Ref.^65^ and explored in Ref.^66^ using one-dimensional hydrodynamic simulations. A possible experimental design is presented in Fig. 10 using realistic parameters, which would enable the observation both of attosecond electron bunches in transmission and their associated HHG in reflection. The phase of electron ejection from the plasma bulk is locked at the electromagnetic field peak of the laser pulse cycle. Those electrons ejected close to parallel to the laser propagation direction will experience Vacuum Laser Acceleration to high energies while retaining their phase and attosecond duration. From Eqs. (6) and (13), the low-density polyethylene target produces larger and more energetic bunches than the aluminium targets used in previous sections and is thus a more practical choice for this experiment.

Note that despite their lower coherence and longer duration, bulk electron bunches would likely be more suited to confirmation of ZVP scaling relationships via a parameter scan, as presented in Fig. 5 of the manuscript, given the simplification to the experimental design and absence of further acceleration phases. Targets must be sufficiently thin to enable electron bunches to escape the target rear but thick enough for the target to maintain its structure over the duration of the laser pulse, a few microns is generally acceptable.

In prior sections of this paper, the laser pulse was focused on the centre of the plasma block, leading to electron bunch detection to the rear and either side of the block. In the arrangement of Fig. 10, the laser pulse is focused onto one edge, maximising laser intensity for electron bunches escaping from this point of interest (these electron bunches have the favourable properties of high charge, low emittance and attosecond duration). A ticker tape, moving out of the plane of Fig. 10 would enable high experimental repetition rates and data collection. In practice, however, experimental conditions do not have the consistency of simulation. Laser-pointing fluctuations, typically on the order of a focal spot, reduce the shot success rate. This can be overcome with the recent emergence of high-repetition-rate petawatt-class facilities which enable gathering of the necessary statistics. Target edges do not typically have the required precision, therefore requiring specialist engineering. This is mitigated by preplasma expansion and the smoothing effect of the interaction as noted by Dromey et al.^67^.

While normal incidence is most convenient for simulations, due to the high intensity of reflected harmonics, experiments must be performed with oblique laser incidence. Suitable shielding must also be provided in the specular direction, with the awareness that the focal length of the relativistic plasma mirror will reduce with time due to hole boring^68^, as can be observed in Supplementary Movie S1, and that the peak reflected intensity at the focus can be over 1000 times that of the incoming laser^69^.

Electron bunch formation due to ponderomotive pressure and charge separation at the plasma surface has been identified in oblique incidence^20,48^, however, the electron bunch energy expressions must be modified. Maximum electron bunch displacement for oblique incidence has been calculated in Ref.^20^. The increase in peak displacement for increasing angle follows from the presence of a component of the laser pulse electric field acting into the plasma bulk, increasing the energy stored in the pseudo-capacitor and therefore also the final electron energy, however, the increase remains on the order of the normal incidence electron bunch energy.

### Conclusion

The Zero Vector Potential mechanism describes the post-ponderomotive rapid absorption of ultra-relativistic laser energy by an overdense, collisionless and fully ionised plasma on sub-laser pulse cycle timescales. In this work, simulations have shown that from currently operational petawatt-class short pulse laser facilities and transversely semi-mass-limited targets, the solid density plasma can withstand the extreme electromagnetic fields of the laser pulse and thus the ZVP mechanism and associated scalings apply, producing in transmission a train of attosecond duration, nano-Coulomb electron bunches, each with a transverse emittance of a few nanometre-radians. Such charge and quality are comparable to state-of-the-art electron bunch accelerators, yet, operating on the attosecond timescales at which atomic processes occur, these electron bunches can be manipulated to literally ‘shed light’ onto fundamental biological and chemical processes. The ZVP energy scaling theory has been extended to the transversely semi-mass-limited case and, by accounting for VLA, has been shown not only to apply to the total scaling for hot plasma electrons of a given simulation but to apply to the absolute energy of individual bunches. This is an important step for the applications of such attosecond electron bunches while also demonstrating the laser pulse cycle independence of the interaction and enabling greater data extraction from each simulation. Via a massive 2D PIC parameter scan the energies of such mass-limited electron bunches have been compared to those predicted by the ZVP model, identifying a range of validity for the model, specifically $a_0$ > 10, *S* > 1. These simulations also confirm the laser pulse ZVP energy absorption scaling in 2D via bulk propagating ZVP electron bunches up to and into the QED regime, demonstrating the role of ZVP energy scalings in determining the QED transition point of the system. The defining characteristics of the ZVP mechanism are identified for the first time in 3D PIC simulation, confirming the anticipated 2D nature of the electron dynamics. The intrinsic link between the CSE theory of high harmonic generation and ZVP theory has been discussed and an experiment design presented to simultaneously observe both phenomena, a step on the path to next-generation bright attosecond diagnostics.

## Methods

### Simulations

The parameter scan simulations were run with the PIC code SMILEI^70^ on the ARCHER2 UK National Supercomputing Service, 2D3V configuration, where particles are confined to 2D but velocities and fields are defined in 3D. Simulation parameters are given in Table 1, such a setup is similar to that which will be possible with the ELI-NP 10-PW beamline^8^. Simulation units are included, these are normalised units relative to $\omega _\mathrm{L}$. For more details see the Smilei documentation^71^.

**Table 1:** Simulation parameters in both real and normalised simulation units for the 2D3V simulations. Solid aluminium has a density of 2.7 ${\hbox {g}}\;{\hbox {cm}}^{-3}$.

Laser (2D, *p*-polarised)		
Parameters	Real	Sim
Wavelength, $\lambda$ (nm)	1060	2$\pi$
Angular frequency, $\omega _\mathrm{L}$ (fs^-1^)	1.8	1
Beam width, $L_0$ (nm)	6$\lambda$	12$\pi$
Focal point, ($x_\mathrm{f}$, $y_\mathrm{f}$) (nm)	(2$\lambda$, 5$\lambda$)	(4$\pi$, 10$\pi$)
Spatial envelope, $E_y$	$E_y \sim e^{-(y-f_y)^2/L_0^2}$	
Temporal envelope, $E_t$	$E_t \sim e^{-(t-4\lambda /c)^2/((4\lambda /3c)^2\ln 2)}$	
Simulation box		
Size, $x \times y$ (nm)	$4\lambda \times 10\lambda$	$8\pi \times 20\pi$
Sim length (fs)	35.22	20$\pi$
Spatial resolution, $\Delta x$ (nm)	$\lambda /128$ = 8.28	0.0491
Temporal resolution, $\Delta t$ (as)	$\Delta x/11c$ = 2.51	0.00446
Plasma (collisionless, fully pre-ionised aluminium plasma)		
Electron *x* profile, *n*(*x*)	$${\left\{ \begin{array}{lll} n_\mathrm{e} \text { for} \; 2\lambda \le x \le 3\lambda ,\\ n_\mathrm{e}e^{(x-2\lambda )/0.2\lambda } \text { for} \; x \le 2\lambda , \\ 0 \text{ otherwise.}\\ \end{array}\right. }$$	
Electron *y* profile, *n*(*y*)	$${\left\{ \begin{array}{ll} 1 \quad \text { for } 2\lambda \le y \le 8\lambda ,\\ 0 \quad \text { otherwise.}\\ \end{array}\right. }$$	
Ion profile, $n_\mathrm{i}$	$n_\mathrm{i} = n(x)n(y)/13$	
Ion charge	13*e*	
Macro-electrons per cell	484	
Macro-ions per cell	25	
Parameter scan		
Plasma density, $n_\mathrm{e}$ (${\hbox {gcm}}^{-3}$)	0.003–7	1–1000
Peak laser *E*-field & $a_0$ (${\hbox {V}}\,{\hbox {m}}^{-1}$)	$10^{10}$–$10^{16}$	0.01–5000

The plasma is initialised as presented in Supplementary Movie S1 and the laser propagates in the $\textbf{x}$-direction, focused normally on the plasma surface. As bunch formation occurs due to coherent electron motion, the greater the uniformity of the target, the stronger the response. The dimensions of the plasma block are of sufficient size to allow observation of effective bunch formation on both sides of the plasma. An exponential preplasma of scale length $0.2\lambda$ is present on the left plasma surface, the side that primarily interacts with the laser. Such a scale length is most efficient for HHG^66^, as the electron bunches are responsible for HHG, it is therefore reasonable to assume that this would also be the most effective for electron bunch formation. Macro-electrons and -ions are initialised regularly for a total of 1.5 $\times$ 10^8^ macro-particles, each representing a chunk of real particles. Since the plasma mirror has a surface length equivalent to the laser beam width, just over 50% of the Gaussian laser pulse energy interacts with the surface. The pulse is cut short around the central 8 optical cycles, for a total pulse duration of 28.3 fs. For the largest values of $a_0$ considered here even those cycles outside the full-width-half-maximum of $8\lambda /3c = 9.4$ fs are ultra-relativistic and therefore lead to coherent bunch generation.

A large parameter space is explored as detailed in Table 1: electron density is scaled from critical to beyond solid density for aluminium and the laser intensity is varied from 10^14^ ${\hbox {W}}\,{\hbox {cm}}^{-2}$ to 10^25^ $\hbox {W}\,{\hbox {cm}}^{-2}$. This captures the transitions from non-relativistic to relativistic, through to ultra-relativistic and then on to the QED regime. The next generation of high-power lasers, such as Shanghai’s SULF^10^, will enable the exploration of the most extreme intensities considered here.

For the 3D simulation, some minor adjustments to the simulation setup were made without significantly altering the dynamics. This was confirmed by comparing it to an equivalent 2D simulation. The target and focal point are translated by $\lambda$ in the $-\hat{\mathbf {x}}$-direction. The number of macro-electrons and -ions per cell are 729 and 8 respectively, corresponding to a reduction in the linear macro-particle density. Particles were initialised randomly to avoid a numerical error relating to the larger surface area of the plasma block in 3D. The target extends $0.75\lambda$ in the $\hat{\mathbf{z}}$-direction. The choice of target size in the *z* direction is somewhat arbitrary given the minimal variation in electron motion in this direction, as demonstrated by Fig. 7, provided the target is of sufficient thickness to maintain the ZVP fields at the front of the target, typically thicknesses should be on the order of the laser pulse wavelength.

The QED processes of radiation reaction (inverse Compton scattering) and non-linear Breit–Wheeler pair production are included for photons and electrons only using the in-built SMILEI packages^70^. Linear Breit-Wheeler pair production can safely be ignored for these simulations: Breit-Wheeler pair production occurs when high energy radiation reaction produced photons travelling in the $-\textbf{x}$-direction interact with the laser pulse. The threshold for linear pair production is given by

18 $$\begin{aligned} E_1 E_2 > (m_\mathrm{e}c^2)^2, \end{aligned}$$

where $E_1$ and $E_2$ are the energies of the two interacting photons. For a laser photon of energy $\hbar \omega$, the high-energy photon must have energy greater than 225 GeV to satisfy this condition. Typical radiation reaction produced photons have energies of the order of the electrons that produced them. Via the ZVP mechanism, those energies are $m_\mathrm{e}c^2a_0/S$. For the simulations conducted, the largest $a_0$ and smallest *S* values were 5000 and 0.25 respectively. Consequently, the peak mean electron bunch energy is 10 GeV and therefore linear Breit-Wheeler pair production is suppressed.

In Ref.^72^, an alternative mechanism for linear Breit–Wheeler pair production is discussed. Here, forward- and back-scattered radiation reaction produced photons interact within a plasma channel to form electron–positron pairs. However, as the setup in this paper produces primarily a surface interaction, such scattered photons do not at any point cross paths and so cannot interact. Therefore, this mechanism will not occur in the simulations presented here.

Particle merging is turned on for the macro-photons in simulations with $a_0 > 1800$. This had a negligible impact on the simulation results but prevented overloading the supercomputer memory due to the vast number of photons produced via radiation reaction.

There is much discussion over the accuracy of the Boris pusher^73^, used in this work to update particle positions, at the extreme laser intensities considered here due to the non-commutative nature of electric field boosts and magnetic field rotations^74,75^. In Ref.^74^ the criterion $c\Delta t / \lambda \ll 1/a_0$ is derived for the case of an underdense plasma, however, the significance of the impact for an overdense plasma such as that considered here has not been determined and therefore caution must be advised. While^75^ presents an accuracy condition in the cubic of the temporal resolution for all plasmas, it is unfortunately not generally valid to use this to obtain a condition such as the one of^74^.

### Transverse emittance

A suitable measure for the quality of an electron bunch is its transverse emittance. A bunch of particles is fully described by its 6-dimensional phase space particle distribution, $\rho (x,p_x,y,p_y,z,p_z)$, where $\textbf{p} = p_x{\hat{\mathbf{x}}}+ p_y{\hat{\mathbf{y}}} + p_z{\hat{\mathbf{z}}}$ is the canonical momentum^76^. The electron bunch under consideration here propagates at a median angle of − 0.393 rad to the *x*-axis. Via a rotation of the coordinate system, one can write the distribution in terms of coordinates aligned with the bunch propagation direction, $\rho (\mathbf {x'},\mathbf {p'}) = \rho (x_\mathrm{L},p_\mathrm{L},x_\mathrm{T},p_\mathrm{T},x_\mathrm {T'},p_\mathrm {T'})$, where L is the direction longitudinal to bunch propagation, *T* is transverse to bunch propagation and in the 2D simulation plane and $\mathrm {T'}$ is transverse but perpendicular to the plane of the simulation, the *z*-direction. Under ideal conditions, the extent of a beam in this phase space, termed the *emittance*, is constant in time and therefore a useful beam parameter. Naturally, for the high charge beam under consideration, there will be space charge growth of the emittance but this can reasonably be ignored on the timescales of interest.

The emittance is in practice split into three parts and calculated by projecting the distribution onto three two-dimensional orthogonal subspaces, corresponding to each of the three directions defined above. The area of each subspace that defines the corresponding emittance is restricted to an ellipse containing the high-density core of the particle distribution in that subspace. For subspace *i*, where $i = \textrm{T}$ or $\mathrm {T'}$, the *transverse normalised emittance* can be derived as^77^

19 $$\begin{aligned} \epsilon ^i_\mathrm{n, rms} = \frac{1}{m_\mathrm{e}c}\sqrt{\langle x_i^2\rangle \langle p_i^2\rangle - \langle x_ip_i\rangle ^2}, \end{aligned}$$

where $\langle \rangle$ defines the second central moment of the particle distribution,

20 $$\begin{aligned} \langle ab \rangle = \frac{\int ab \rho (\mathbf {x'},\mathbf {p'}) \textrm{d}V}{\int \rho (\mathbf {x'},\mathbf {p'}) \textrm{d}V}- \frac{\int a \rho (\mathbf {x'},\mathbf {p'}) \textrm{d}V\int b \rho (\mathbf {x'},\mathbf {p'}) \textrm{d}V}{(\int \rho (\mathbf {x'},\mathbf {p'}) \textrm{d}V)^2}, \end{aligned}$$

here $$\textrm{d}V = \Pi _i \textrm{d}x_i\textrm{d}p_i$$.

Most often when working with emittances, it is the *transverse geometric emittance*, $\epsilon ^i_\mathrm{rms}$ that is referred to, as this can be more easily measured in experiments^76^. The two are related via

21 $$\begin{aligned} \varepsilon ^i_\mathrm{rms} = \frac{\varepsilon ^i_\mathrm{n, rms}}{\gamma \beta _\mathrm{L}}, \end{aligned}$$

where $\gamma = 1/\sqrt{1-\beta ^2}$ and $\beta _\mathrm{L} = v_\mathrm{L}/c \approx 1$ as all bunch electrons are ultra-relativistic. Taking the mean $\gamma$ from Fig. 1c, $\gamma = (98.6$ ± 20.5), while for the 3D simulation electron bunch, $\gamma = (115$ ± 13).

The $(x_\mathrm{T},p_\mathrm{T})$ phase space is presented in Fig. 1d. The ellipse plotted describes the area associated with the transverse normalised emittance, $A = \pi \epsilon ^{\textrm{T}}_\mathrm{n, rms}$, and can be calculated from the appropriate Courant-Snyder parameters^77^.

Caution must be advised in applying the standard definition of the emittance to non-Gaussian beam distributions, as can be observed in Figs. 7 and 1d, the emittance can be over- or underestimated. While for an ideal Gaussian distribution, the elliptical contour defining the emittance contains 39.3%^76^ of the total population of the beam, the elliptical contours of Figs. 7 and 1d contain 38.6% and 75.5% of bunch electrons respectively. The long low-density tail of the distribution in Fig. 1d is responsible for the overlarge contour calculated.

### Supplementary Information

Supplementary Information.

## Data Availability

Data sets generated during the current study are available from the corresponding author on reasonable request.
